# Amino Acid Metabolism and Transport Mechanisms as Potential Antifungal Targets

**DOI:** 10.3390/ijms19030909

**Published:** 2018-03-19

**Authors:** Matthew W. McCarthy, Thomas J. Walsh

**Affiliations:** 1 Departments of Medicine, Weill Cornell Medicine of Cornell University, New York, NY 10065, USA; thw2003@med.cornell.edu; 2 Departments of Pediatrics, and Microbiology & Immunology, Weill Cornell Medicine of Cornell University, New York, NY 10065, USA

**Keywords:** amino acid transporters, metabolism, antifungal targets, cispentacin, icofungipen, sinefungin

## Abstract

Discovering new drugs for treatment of invasive fungal infections is an enduring challenge. There are only three major classes of antifungal agents, and no new class has been introduced into clinical practice in more than a decade. However, recent advances in our understanding of the fungal life cycle, functional genomics, proteomics, and gene mapping have enabled the identification of new drug targets to treat these potentially deadly infections. In this paper, we examine amino acid transport mechanisms and metabolism as potential drug targets to treat invasive fungal infections, including pathogenic yeasts, such as species of *Candida* and *Cryptococcus*, as well as molds, such as *Aspergillus fumigatus*. We also explore the mechanisms by which amino acids may be exploited to identify novel drug targets and review potential hurdles to bringing this approach into clinical practice.

## 1. Introduction

Recent advances in our understanding of the fungal life cycle, functional genomics, and proteomics have enabled the identification of multiple potential new drug targets that could bolster the arsenal of available options to treat invasive fungal infections (IFIs), which include pathogenic yeasts, molds, and thermally-dimorphic fungi [1,2,3]. These conditions present an expanding public health threat due to a rise in the frequency of IFIs, and an increasing resistance to standard antifungal therapy, and because no new classes of antifungal agents have been approved since 2006 [4,5,6].

Three major classes of antifungal agents are currently available to clinicians to treat IFI: (1) the polyene amphotericin B-deoxycholate (its lipid and liposomal formulations), which binds fungal cell membrane ergosterol leading to cell lysis; (2) azoles that inhibit ergosterol biosynthesis (fluconazole, itraconazole, voriconazole, posaconazole, isavuconazole); and (3) echinocandins (caspofungin, micafungin, anidulafungin) that inhibit fungal (1→3)-β-d-glucan cell wall biosynthesis [7,8]. In many cases, however, these agents carry drug- or class-specific toxicities, have major drug interactions, or are not sufficiently active because fungi have become resistant, underscoring the need for novel agents and drug targets [9,10,11].

Amino acids constitute a major nutritional source for fungi, and elements of their metabolic pathways and transport may be utilized as targets for new antifungal agents [12]. Yeasts and molds, such as species of *Aspergillus*, possess multiple amino acid transporters that are classified according to a variety of factors, including structure, subcellular location, substrate specificity range, and regulation [13,14]. When comparing the transporters of three completely sequenced eukaryotic genomes—*Saccharomyces cerevisiae*, *Arabidopsis thaliana*, and *Homo sapiens*—amino acid transporter types can be distinguished according to transport mechanism, phylogeny, substrate spectrum, and cell specificity [15].

Conformations adopted by some of these transporters in response to amino acid binding appear crucial to promote ubiquitin-dependent endocytosis and trigger signaling responses that may serve as potential drug targets [16,17,18]. Below, we review novel compounds that interfere with amino acid transport, especially those that interact with tRNA synthetase, with special attention paid to those that are active against human fungal pathogens, including pathogenic yeasts and molds (Table 1).

## 2. Yeasts

### 2.1. Icofungipen

**Figure 1:**
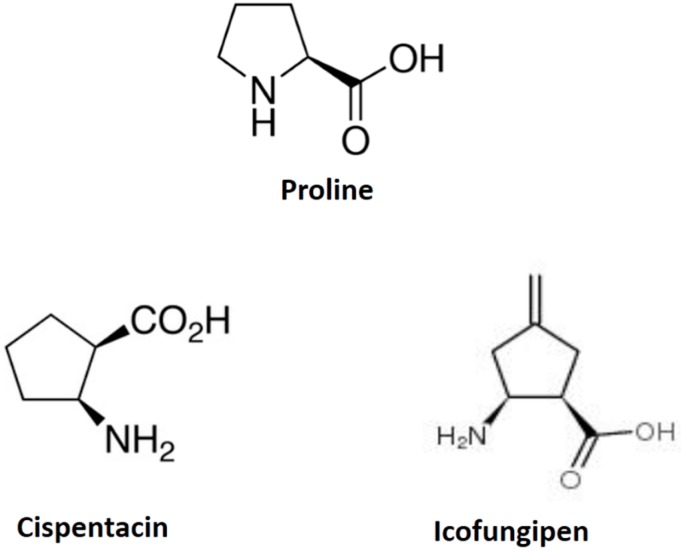
Comparative structures of proline, cispentacin, and icofungipen.

Beta-amino acids, including icofungipen, have been identified as potential new antifungal compounds to treat yeast infections, such as candidiasis [19]. Icofungipen, formerly known as PLD-118 (which was formerly known as BAY 10-8888), was isolated from the culture broth of a strain of *Bacillus cereus*, and is water-soluble and amphoteric (Figure 1) [20,21]. The mode of action is due to an active transport of the molecule via proline and other amino acid permeases into fungal cells, where it inhibits the isoleucyl-tRNA synthase and protein synthesis [22,23,24,25].

Initial studies indicated that icofungipen displayed minimal in vitro activity against medically-relevant fungi, but good activity against *Candida albicans* A9540 in a murine model [26]. Subsequent work revealed that the drug interferes with fungal amino acid transport, synthesis, and cellular regulation of amino acid metabolism [27,28,29]. The 50% inhibitory concentration (IC50) and IC100 values of icofungipen against clinical isolates of *Candida albicans* were in the ranges 6.3 approximately 12.5 and 6.3 approximately 50 micrograms/mL, respectively, and demonstrated good efficacy in mice inoculated with *Candida* spp. by both parenteral and po administrations [30]. There was also strong activity against *Cryptococcus neoformans* and in both vaginal and pulmonary infections, and the drug did not demonstrate substantial toxicity in a murine model [21,31].

Icofungipen activity against species of *Candida* has been demonstrated in vivo. Walsh and colleagues evaluated the compound in the treatment of experimental subacute disseminated candidiasis in persistently neutropenic rabbits [32]. The drug was administered one day after the intravenous inoculation of *C. albicans* blastoconidia, and was continued for ten days. For these experiments, New Zealand white rabbits were treated with icofungipen at 4 (ICO-4), 10 (ICO-10), and 25 (ICO-25) mg/kg of body weight/day; other rabbits were treated with fluconazole at 10 mg/kg/day; another set of rabbits were treated with amphotericin B at 1 mg/kg/day; the final group consisted of untreated controls. Rabbits treated with ICO-10 (*p* < 0.01) and ICO-25 (*p* < 0.001) showed significant dosage-dependent tissue clearance of *C. albicans* in comparison to untreated controls, and icofungipen demonstrated consistent pharmacokinetics (PK) throughout the series of experiments. In plasma, PK approximated a dose-dependent relationship. There was no significant hepatotoxicity or nephrotoxicity in icofungipen-treated rabbits. The drug followed dose-dependent PK, and was proved to be effective in the treatment of experimental disseminated candidiasis in immunosuppressed, neutropenic rabbits, including those with experimental meningoencephalitis [33].

Similar to the transport of natural amino acids in the yeast *Saccharomyces cerevisiae*, the transport of icofungipen into the cell is unidirectional [22]. Inside the fungal cell, the compound inhibits isoleucyl-tRNA synthetase, resulting in inhibition of protein synthesis and cell growth [34]. As expected, intracellular isoleucine reverses icofungipen-induced growth inhibition [35]. The molecule inhibits isoleucyl-tRNA synthetase in a similar manner, with the same concentration dependency curve, assuming nearly 200-fold accumulation in protein biosynthesis.

In one study, *C. albicans* cells accumulated icofungipen intracellularly to a concentration about 200 that in the medium when grown in media with a variety of nitrogen sources [22]. Uptake was mediated by an H^+^-coupled amino acid transporter with specificity for branched-chain amino acids (isoleucine, leucine, and valine) and showed a KT (Michaelis constant of the transport reaction) of 0.95 mM [22,36]. These were the basis for subsequent in vivo studies, which will be reviewed below.

The drug was evaluated in escalating dosages against experimental invasive candidiasis (both esophageal and oropharyngeal models of disease) caused by fluconazole-resistant *C. albicans* in rabbits with immune impairment. These rabbits were divided in a manner similar to other studies involving invasive candidiasis: some rabbits received icofungipen at 4, 10, 25, or 50 mg/kg of body weight/day via intravenous (iv) twice daily injections; other rabbits received FLC at 2 mg/kg/day via iv twice daily injections. Another group received amphotericin B (DAMB) iv at 0.5 mg/kg/day, and these were all compared to untreated controls to assess dose-dependent antifungal activity.

Icofungipen- and DAMB-treated animals revealed a dosage-dependent clearance of *C. albicans* from the tongue, oropharynx, and esophagus that proved to be statistically significant as compared to untreated controls (*p* ≤ 0.05, *p* ≤ 0.01, *p* ≤ 0.001, respectively), while fluconazole had no significant activity, while the safety profile was similar to that of fluconazole. The work demonstrated dosage-dependent antifungal activity of icofungipen in the treatment of experimental drug-resistant oropharyngeal and esophageal candidiasis.

Possible metabolism of the drug by rat, dog, and human S9 liver homogenates, and inhibition of human cytochrome P450 (CYP) enzymes have also been investigated [37]. In a study by Parnham and colleagues, CYP assays were performed using pooled human liver microsomes with substrates selective towards human CYP1A2, CYP2A6, CYP2B6, CYP2C9, CYP2C19, CYP2D6, CYP2E1, and CYP3A [38]. Encouragingly, the compound did not inhibit any of the CYPs tested, suggesting little likelihood for interaction of the compound with drugs metabolized by these enzymes. Icofungipen has been in phase II clinical trials for the treatment of candida infections, but this research has been discontinued. In human toxicity studies, suppression of spermatogenesis in male volunteers was observed as a possible off-target adverse event. “It is concerning that good activity in vivo did not correspond to the potency in in vitro models for proline analogues of icofungipen and cispentacin (The structural and physiologic differences between cispentacin and icofungipen are almost negligible). This issue needs to be addressed by either modification of the media (agarose vs agar), amino-acid dropouts, temperature, or some other variable”. Nonetheless, other compounds that affect amino acid transport may serve as therapeutic options for the treatment of invasive mycoses, especially those to address invasive mold infections, which will be reviewed below.

### 2.2. Inositol Pathway

The genus of the yeast-like fungus *Pneumocystis* is comprised of obligate fungal pathogens that live only in the lungs of their mammalian hosts [39,40,41]. The fact that the organism does not routinely grow in culture indicates that some crucial nutrients or amino acids are missing in culture media. Metabolic studies indicate that the fungus does not synthesize ergosterol, and has to scavenge cholesterol from its host, while genome-sequencing studies have noted that the organism has dramatic underrepresentation of amino acid synthesis pathways [42,43,44].

Porollo and collaborators conducted a genome analysis of several sequenced species of the *Pneumocystis* genus, including *P. jirovecii*, *P. carinii*, and *P. murina*, as well as the fission yeast *Schizosaccharomyces pombe* [45]. Their study revealed an overexpression of the inositol phosphate metabolism pathway in the *Pneumocystis* relative to that found in *S. pombe*. Addition of inositol in a primary in vitro culture system substantially enhanced the viability of *Pneumocystis* throughout a two week period, but in and of itself, was not sufficient, suggesting that these fungi are inositol auxotrophs and that this molecule is a crucial nutritional requirement needed for culture, but in and of itself, is not sufficient for continuous cultivation [46]. This revelation also supports another important finding: exogenous sources of inositol provide a new fungal drug target [47]. The work by Porollo and others demonstrates that the strategy of comparative genomics holds the tremendous promise to identify all of the essential nutrients and amino acids that fungi require to live, and that this method might be utilized to identify new treatment options to address medical mycoses.

## 3. Molds

### 3.1. Histidine Pathway

*Aspergillus fumigatus* is an opportunistic pathogen that is the most prevalent airborne fungal pathogen causing invasive fungal infections in patients with immune impairment [48,49,50]. Dietl and colleagues recently exploited the histidine biosynthetic pathway to demonstrate its suitability as a potential antifungal target to treat aspergillosis [51]. This amino acid synthesis pathway has emerged as an attractive target because the histidine metabolic pathway is found in a diverse group of microorganisms and plants, but is absent in mammals [52,53]. Their team showed that a gene encoding imidazoleglycerol-phosphate dehydratase (HisB) in pathogenic mold causes decreased resistance to starvation.

This work has important implications for antifungal drug development [4]. The work by Dietl and colleagues underscores the limited histidine availability in *A. fumigatus* host niches, and highlights the histidine biosynthetic pathway as being an attractive target for development of novel antifungal therapy approaches [51]. Further studies of the transporters for histidine precursors may also elucidate another antifungal therapeutic target.

### 3.2. Galactofuranose Pathway

Galactofuranose (Galf) is the 5-member-ring form of galactose found in the walls of many pathogenic molds, including species *Aspergillus*, but it is not in mammals [54]. Galf is found in many medically-relevant mycoses, indicating that Galf is an important component for survival and reproduction [55,56]. Interestingly, Galf has never been found humans, thus Galf-biosynthetic pathways have raised much interest as targets for drug development as they pertain to amino acids and their transport [57,58,59,60,61]. UDP-galactofuranose mutase (UgmA) generates UDP-Galf from UDP-galactopyranose (UDP-Galp, 6-member ring form) in cytoplasm, so the UDP-Galf residues it generates must be moved into an endomembrane compartment prior to inclusion into cell wall components [62].

Based on its high amino acid sequence homology, the gene UgtA was identified by Afroz and collaborators to encode the UDP-Galf transporter in species of pathogenic mold [63]. Their group noted that the ugtAΔ protein that was expressed resembled that of ugmAΔ, which had wide, compact colonies, angular hyphae, and attenuated sporulation. Like ugmAΔ, the ugtAΔ hyphal walls were much thicker than other strains. AfglfB restored growth and reproduction in the ugtAΔ strain, revealing that these proteins have homologous function. Fluorescent staining with EBA2 indicated that ugtAΔ hyphae failed to produce Galf, but was recapitulated in the AfglfB-complemented strain.

Compared to wild type strains, spore production for ugtAΔ was reduced one hundred-fold and spore germination was reduced to half. UgtAGFP had a punctate distribution in hyphae, phialides, and young spores. Notably, the ugtAΔ strain was significantly more sensitive than wild type to caspofungin, which inhibits (1→3)-β-d-glucan synthesis, suggesting that drugs that could be developed to target UgtA function. 

Since Galf is not biosynthesized by higher eukaryotes, such as mammals, the molecule is also an attractive candidate for diagnosis of fungal infection. A monoclonal antibody that recognizes Galf is commercialized for detection of aspergillosis [64]. In theory, these compounds could be used for the synthesis of artificial carbohydrate-based antigens, and may play an important role in both diagnosis and treatment of invasive fungal infections.

## 4. Drug Discovery and Delivery

### 4.1. Drug Discovery

Thus far, we examined amino acid transport mechanisms and metabolism as a potential drug target to treat invasive fungal infections. However, amino acids and their metabolites may also be exploited to identify novel drug targets. For example, the yeast-like fungus *Pneumocystis jirovecci* is dependent upon *S*-adenosylmethionine (AdoMet), a key metabolic intermediate with a wide range of functions, including protein and nucleic acid methylation, phospholipid synthesis, folate metabolism, polyamine synthesis, and the production of glutathione [65,66,67,68]. *P. jirovecci* possess high-affinity, highly selective AdoMet transporters, and the dependence on this metabolite is supported by animal studies: PCP attenuates AdoMet in lung tissue and depletes plasma AdoMet [41,69]. This requirement of exogenous AdoMet suggests transport as a potential drug target. 

**Figure 2:**
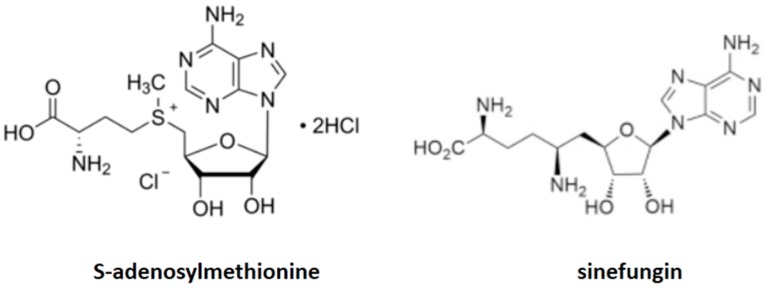
Comparative structures of *S*-adenosylmethionine and sinefungin.

Perez-Leal and colleagues reported on the discovery of *PcPET8*, a *P. jirovecci* gene with homology to mitochondrial AdoMet transporters. When expressed by yeasts such as *Saccharomyces cerevisiae*, the translated protein locates properly to the mitochondrion and complements a strain of *S. cerevisiae* lacking its native mitochondrial AdoMet transporter [70]. The importance of AdoMet transport is revealed by the ability of the AdoMet analogue sinefungin to block the uptake of *P. jirovecci* AdoMet and inhibit growth in culture (Figure 2) [66]. Because PcPET8 is critical for *P. jirovecci*, the yeast construct has potential as a surrogate for testing compounds against *P. jirovecci*, and potentially, other fungi.

The antimicrobial agent sinefungin is active against *P. jirovecci* in culture, and is known to compete with AdoMet for *S. cerevisiae* PET8 transport [71]. It is the only compound Perez-Leal and colleagues found able to block the uptake of AdoMet by *P. jirovecci*. Their data indicate that 100 mM of sinefungin blocks the uptake of AdoMet by 90%, and completely inhibits growth in tissue culture, a finding that supports AdoMet transport as a drug target. However, toxicology studies in animals suggest that the drug can cause severe nephrotoxicity, limiting is use as a drug candidate in humans. *P. jirovecci* AdoMet uptake is more susceptible to sinefungin than *S. cerevisiae*. This suggests that AdoMet transport and metabolism may serve as a novel drug target. Although adverse reactions make sinefungin an unlikely drug candidate—it displayed substantial nephrotoxicity in goats—the ability of this compound to inhibit the growth of fungi suggests that this construct may prove useful as a tool to identify other compounds that block AdoMet transport in other pathogenic fungi, and may prove helpful in identifying novel drug candidates [1,72].

### 4.2. Drug Delivery

Amino acid permeases (AAPs) in the cell membrane of fungi such as *S. cerevisiae* are responsible for the uptake of proteins, amino acids, and other small molecules involved in regulation of their cellular levels [73]. Basic amino acids selectively accumulate in vacuoles, but hydroxylated amino acids are almost excluded from them, indicating that this location might be preferentially exploited for drug delivery [13]. Auxin permeases involved in the vacuolar compartmentalization of amino acids have been recently identified in studies using *S. cerevisiae* [73].

Using in silico analyses, one group found that the C-terminal sequences have qualities that differentiate them from the termini of amino acid transporters in other organelles, and that this may be exploited for drug delivery [74]. Popov-Čeleketić and colleagues showed that one sequence, referred to as PM_asseq_, contains an amino acid signature that leads to different localization patterns. This disruption of sequence has an adverse effect on cell viability, which is independent of the role played by amino acid transporters [75]. They argue that PM_asseq_ modulates the function and localization of AAPs along the PM, which could, in theory, be used as a delivery vehicle for the PM.

This property is just one way that permeases have been utilized. FTY720 is a sphingoid base analog that acts as an anticancer agent in animal models [76]. Its activity against tumor cells is attributed to its ability to trigger endocytosis of several nutrient transporters, which, in turn, rely on amino acid transport [77]. The observation that FTY720 diminishes production of amino acid permeases in fungi indicates that that the cellular mechanisms it targets are evolutionarily conserved [78]. Barthelemy and colleagues demonstrated that adding FTY720 to yeast cells results in rapid inhibition of the intrinsic activity of multiple amino acid permeases, and reveal that this effect is associated with inhibition of the TORC1 kinase complex, which in turn promotes ubiquitin-dependent permease endocytosis [79]. The group also showed that FTY720 promotes endocytosis of the LAT1/SLC7A5 amino acid transporter in HeLa cells, suggesting that TORC1 deactivation results from FTY720-mediated inhibition of membrane transport. These data indicate that the anti-tumor effect of FTY720 may be due to transporter endocytosis and this mechanism might be utilized for drug delivery in yeasts [77,80,81,82].

## 5. Conclusions and Future Directions

The widespread use of immunosuppressants, hematopoietic stem cell transplantation and solid organ transplantation, and novel immunomodulators in clinical practice, has led to an expanding population of patients who are at risk for invasive fungal infection. The emergence of antifungal drug resistance has added to the substantial morbidity and mortality associated with mycotic infection, and novel therapeutic strategies are urgently needed. Most antifungal drugs either kill a range of fungal pathogens in vitro or considerably slow their growth, but they are not always clinically useful [11].

Amino acids constitute a major nutritional source for fungi and, as noted above, their transport and metabolism may be exploited as potential drug targets. Studies of pathogenic species, such as *A. nidulans* and *S. cerevisiae*, have revealed that they harbor numerous amino acid transporters [83,84,85]. These transporters are now classified according to protein fold in addition to sequence criteria, and differ in substrate, organelle, and regulation, and we have reviewed how these structures might be manipulated to identify novel drug targets.

Discovering new drugs for treatment of resistant fungal infections will always be a challenge because fungi and eukaryotes are so closely related (eukaryotes and far more closely related to humans than are bacteria or viruses). Despite the challenges, opportunities do exist for development of novel therapies. Recent advances in our understanding of the fungal life cycle, functional genomics, proteomics, and gene mapping have enabled the identification of multiple potential new drug targets that could bolster the arsenal of available options to treat resistant fungal infections. In this paper, we have examined targets involved in amino acid transport, and describe how promising new therapies might be developed, with special attention paid to molecules that promote growth inhibition.

As noted above, many drugs with antifungal properties have undesirable side effects or are ineffective against new or reemerging mycoses [86]. Considerable work has been performed to evaluate and exploit amino acid transport mechanisms to identify novel antifungal agents. However, many of these molecules have not found a place in clinical practice due to adverse events, lack of investment, or limited spectrum. Further work is necessary to realize the promise of this approach [2,72].

We believe that investigators should build on existing work to identify compounds that may potentially be useful; for example, there is some evidence that *N*-acetylcysteine may inhibit germination of conidia, and play a role in the disruption of fungal amino acid transport. The scope of this work is limited, but we believe it should be expanded. Medicinal chemistry has been used to identify a variety of novel antifungal agents involving amino acid transport, but there is more work to be done to meet the needs of the expanding population of patients at risk for fungal infections.

It is important for the international community to meet this challenge by bolstering support for research, considering alternative approval pathways, repurposing existing agents that are used to treat other conditions, strengthening ties between academia and industry, and providing financial incentives for the development of new therapeutic options that involve amino acid transport.

As we have noted throughout the manuscript, new treatment options for fungal infections are urgently needed, but it remains a challenge to bring novel agents to the market. The discovery of a new therapy is difficult, high throughput screens are labor intensive, traditional regulatory requirements are stringent, and the financial reward for investigating amino acid transport mechanisms may be limited. Nevertheless, we must continue to investigate these diseases to meet the needs of an expanding population of vulnerable patients.

## Figures and Tables

**Table 1:** Selected Amino Acid Transporters and Potential Pharmacological Targets of Antifungal Therapy.

Amino Acid Transporter	Antifungal Agent	Organism	Structures
Proline	cispentacin (1*R*,2*S*)-2-aminocyclopentane-1-carboxylic acid	*Candida albicans Cryptococcus neoformans*	Figure 1
Proline	icofungipen (1*R*,2*S*)-2-amino-4-methylidene-cyclopentane	*Candida albicans Cryptococcus neoformans*	Figure 1
Mitochondrial *S*-adenosylmethionine transporter	Sinefungin (2*S*,5*S*)-2,5-diamino-6-[(2*R*,3*S*,4*R*,5*R*)-5-(6-aminopurin-9-yl)-3,4-dihydroxyoxolan-2-yl]hexanoic acid	*Pneumocystis Saccharomyces cerevisiae*	Figure 2
